# Outcome and Genetic Factors in IgG4-Associated Autoimmune Pancreatitis and Cholangitis: A Single Center Experience

**DOI:** 10.1155/2017/6126707

**Published:** 2017-03-02

**Authors:** Matthias Buechter, Paul Manka, Falko Markus Heinemann, Monika Lindemann, Benjamin Juntermanns, Ali Canbay, Guido Gerken, Alisan Kahraman

**Affiliations:** ^1^Department of Gastroenterology and Hepatology, University Hospital Essen, Essen, Germany; ^2^Division of Transplantation Immunology and Mucosal Biology, King's College London, London, UK; ^3^Institute of Transfusion Medicine, University Hospital Essen, Essen, Germany; ^4^Department of General, Visceral, and Transplantation Surgery, University Hospital Essen, Essen, Germany

## Abstract

*Introduction*. Most investigations on autoimmune pancreatitis (AIP) were published on Asian cohorts while those on Caucasians are limited. However, there might be differences related to the origin. *Patients and Methods*. We analyzed 36 patients and compared type 1 (AIP1) with type 2 (AIP2). *Results*. The majority of patients suffered from AIP1 (55.6%). AIP1 patients were significantly older than AIP2 patients (54.4 versus 40.8 years). Moreover, 85.0% of AIP1 patients had concurrent autoimmune cholangitis (AIC) while 18.8% of AIP2 patients suffered from overlap to ulcerative colitis (UC). However, AIP1 patients revealed a cholestatic course and had significantly higher immunoglobulin G4 levels (IgG4). When compared to allele frequencies in healthy controls, in patients with AIP1 HLA-B8 reached statistical significance. Response to steroids was excellent in both groups, but we noticed high rates of relapse especially in AIP1 patients. Finally, 3 patients with AIP1 were diagnosed with cholangiocellular carcinoma (CCC). *Conclusion*. In contrast to Asian studies, we found an almost equal distribution of AIP1 and AIP2 patients in our German cohort. AIP2 patients were younger and mostly of female gender whereas AIP1 patients revealed higher IgG4 levels and involvement of the biliary tract in sense of IgG4-associated cholangitis.

## 1. Introduction

Autoimmune pancreatitis (AIP) is a novel and challenging entity. During the last decade, the number of diagnosed patients with AIP has increased worldwide [1, 2]. It is estimated that approximately 5% of all patients with chronic pancreatitis may suffer from AIP [3, 4]. However, it is more likely that the increasing recognition of this disease is due to its increased awareness rather than a true increase in incidence [5, 6].

The International Diagnostic Criteria for AIP published by the “International Association of Pancreatology” in 2011 can be considered as an essential advantage offering uniform and internationally accepted diagnostic rules by summarizing diverse pre-existing guidelines. Shimosegawa and colleagues differentiated between two subtypes, namely, autoimmune pancreatitis type 1 (AIP1) and autoimmune pancreatitis type 2 (AIP2), based on five cardinal features: imaging of the pancreatic parenchyma and duct, serology, other organ involvement, pancreatic histology, and finally, response to steroid treatment [7]. Each feature was categorized as level 1 and 2 findings depending on the diagnostic reliability, making the diagnosis of AIP1 and AIP2 definitive or probable. AIP1 can be distinguished as a pancreatic manifestation of a systemic, immunoglobulin G4- (IgG4-) associated disease with histological correlation with lymphoplasmacytic sclerosing pancreatitis (LPSP), extrapancreatic organ involvement (e.g., IgG4-associated cholangitis, tubulointerstitial nephritis, and retroperitoneal fibrosis), and infiltration with IgG4-positive plasma cells, whereas AIP2 is histologically designated as an idiopathic duct centric pancreatitis (IDCP) with characteristic granulocytic epithelial lesions (GELs). AIP2 rarely shows increased IgG4 levels but is often associated with inflammatory bowel disease (IBD) [8, 9]. Due to the lack of biochemical markers—in few cases—determination of carbonic anhydrase-II antibodies may help in diagnosing AIP2. However, histology by pancreatic biopsy is an important diagnostic step [10].

Nevertheless, AIP is still a diagnostic puzzle and often difficult to diagnose and distinguish. Due to clinical and radiological similarities but a completely different prognosis and therapy, it is of fundamental importance to differentiate between pancreatic cancer and autoimmune pancreatitis. Nearly 30% of patients undergoing surgery for initially suspected pancreatic cancer turned out as benign pancreatic disease afterwards. Therefore, serological markers such as high IgG4 and CA 19-9 levels as well as the presence of carbonic anhydrase-II antibodies might be helpful diagnostic tools [11, 12].

Currently, many investigations on Asian cohorts, especially from Japan, were published while those on Caucasian cohorts are still deficient. It has been suggested that there might be significant clinical differences between patients according to their origin. In contrast to western countries, where AIP2 is supposed to be more frequent (up to 50%), Asian studies with large patient numbers demonstrated that nearly all patients (>90%) suffered from AIP1 [13–15]. In terms of possible etiological factors of AIP, the higher infection rate with *Helicobacter pylori* (HP) in Asian populations might be a possible explanation for this observation. Due to molecular mimicry between HP-carbonic anhydrase and human carbonic anhydrase-II, helicobacter may induce autoimmune pancreatitis.

In the current study, our aim was (i) to compare patients with autoimmune pancreatitis type 1 and type 2 and (ii) to compare our Caucasian cohort with Asian cohorts, especially in terms of clinical course and biochemical markers.

## 2. Patients and Methods

We retrospectively analyzed 36 patients treated for AIP at the Department of Gastroenterology and Hepatology, University Hospital of Essen, Germany, between 2010 and 2016. All patients fulfilled the International Diagnostic Criteria for AIP. In case of uncertain diagnosis according to serology or imaging, AIP was proven histologically in the form of resected pancreatic specimen, pancreatic biopsy via endosonography-guided fine-needle aspiration, or biopsy of the papilla of Vater, showing typical histological features of granulocytic infiltration of the duct wall in attendance of IgG4-positive cells [7].

We compared patients with AIP1 to those with AIP2 with regard to clinical and serological differences and surveyed the following characteristics: origin, gender, age, body mass index (BMI), concurrent autoimmune cholangitis (AIC), overlap to ulcerative colitis (UC), development of malignancies, response to steroid treatment, rate of relapse, immunosuppressive regimen, and profiles of serological and genetic markers.

AIC was diagnosed on the basis of the clinical diagnostic criteria of IgG4-related sclerosing cholangitis published in 2012 by Ohara et al.: (1) characteristic biliary imaging findings, (2) elevation of IgG4 concentrations (>1.350 mg/l), (3) coexistence of AIP, and finally, (4) characteristic histopathologic features [16]. Diagnosis of UC was established by endoscopy revealing abnormalities of the colonic mucosa. Human leukocyte antigen (HLA) typing was performed after previous informed consent of the patients. Disease relapse was defined as the development of symptoms (e.g., abdominal pain and jaundice) and concurrent imaging findings or liver test abnormalities consistent with a new or worsening inflammatory process [10]. Diagnosis of malignancy was proven either by percutaneous liver biopsy or by histological specimen during surgery.

Statistical analysis was performed using Student's *t*-test for variables with normal distribution and Mann-Whitney *U* test for variables without normal distribution. HLA phenotypes were compared using contingency tables. A *p* value of <0.05 was considered as statistically significant. All patients provided written informed consent before enrolment. The study was conducted in accordance with the ethical guidelines of the 2008 Helsinki Declaration, and the protocol was approved by the local ethics committees of the University Hospital of Essen.

## 3. Results

### 3.1. Patient Characteristics

**Figure 1:**
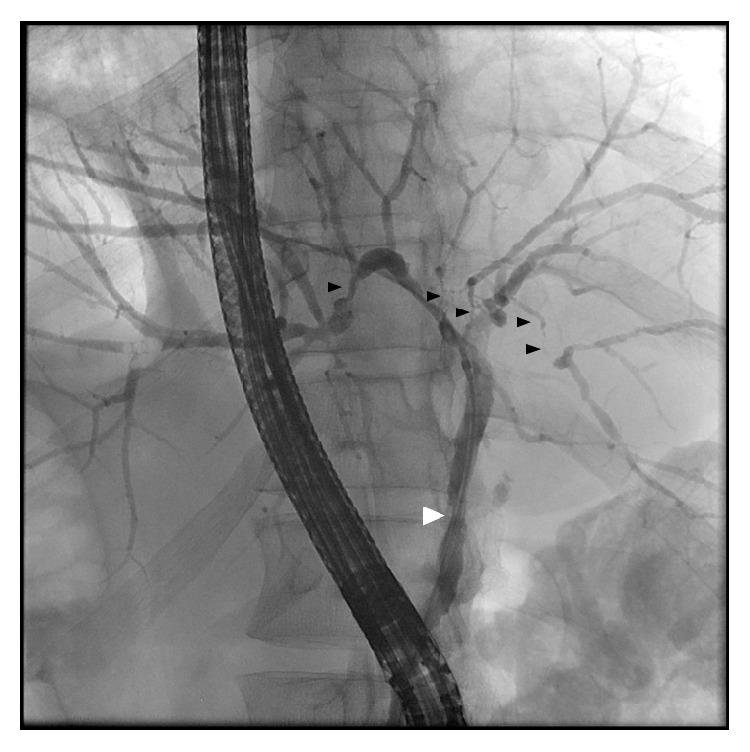
ERC of a patient with IgG4-associated autoimmune cholangitis showing intra- (black arrows) and extrahepatic strictures (white arrow) of the biliary tract (mixed type).

**Figure 2:**
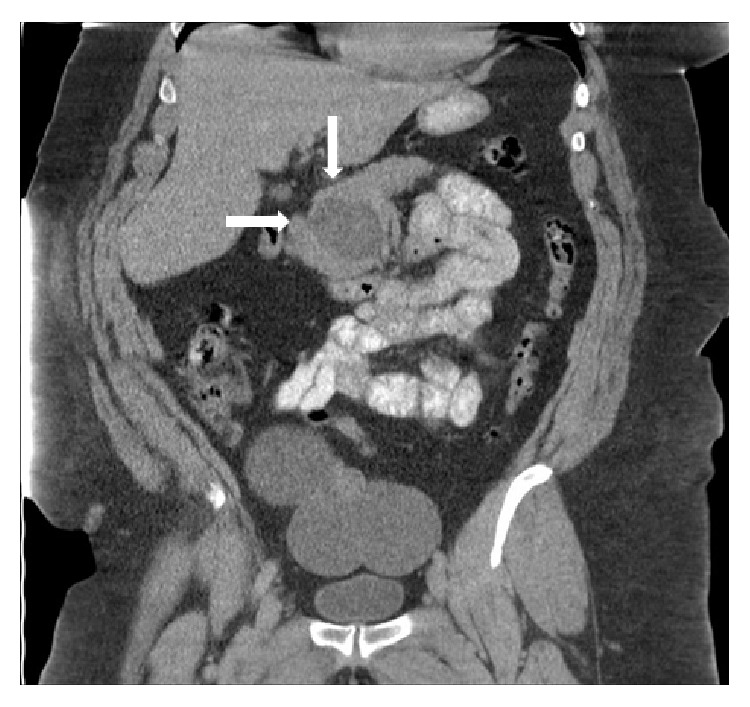
MRI of a patient with autoimmune pancreatitis in coronal orientation showing a tumor in the pancreatic head (focal type; white arrows).

A total of 36 patients were included in the present study (Table 1). All patients of our cohort were of Caucasian origin. The majority of patients suffered from AIP1 (55.6% versus 44.4% with AIP2). Patients with AIP1 were, in general, older (mean age 54.4 ± 4.3 years) than those with AIP2 (mean age 40.8 ± 2.7 years; *p* < 0.05). The majority of AIP1 patients were male (75.0%) while AIP2 patients were mostly of female gender (68.8%). However, 85.0% of the AIP1 patients had concurrent AIC (0% in AIP2; *p* < 0.01) while 18.8% of the AIP2 patients suffered from UC (5.0% in AIP1, *p* = n.s.). In the presence of AIC, the location in patients with AIP type 1 was the following: intra- and extrahepatic involvement (mixed) with 11/17 (64.7%), intrahepatic involvement with 4/17 (23.5%), and extrahepatic involvement with 2/17 (11.8%). Diagnosis of AIC was confirmed by either MRC/P or ERC/P or the combination of these procedures (Figure 1). In case of isolated extrahepatic involvement of the biliary tract (2 patients), discrimination between the primary (IgG4-related) or secondary cholangitis (due to compression by the inflamed pancreas) was retrospectively not possible. There were no significant differences between AIP1 and AIP2 patients with regard to BMI (mean BMI in AIP1 24.9 kg/m^2^ versus 27.0 kg/m^2^ in AIP2; *p* = n.s.). Moreover, 22/36 patients demonstrated pathological changes in pancreatic morphology verified by either MRC/P (14 patients), EUS (6 patients), or CT (2 patients). Among these 22 patients, 14 (63.6%) demonstrated focal involvement and 8 (36.4%) diffuse pancreatic involvement (Figure 2).

### 3.2. Laboratory Parameters

**Figure 3:**
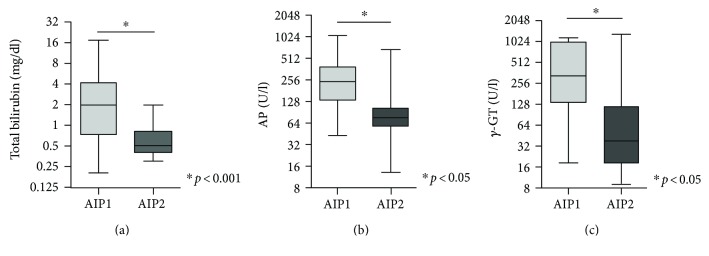
Cholestatic liver enzymes in patients with AIP1 and AIP2. (a) Total bilirubin is significantly increased in patients with AIP1 as compared to patients with AIP2. Likewise, AP (b) and γ-GT (c) were significantly elevated in patients with AIP1.

**Figure 4:**
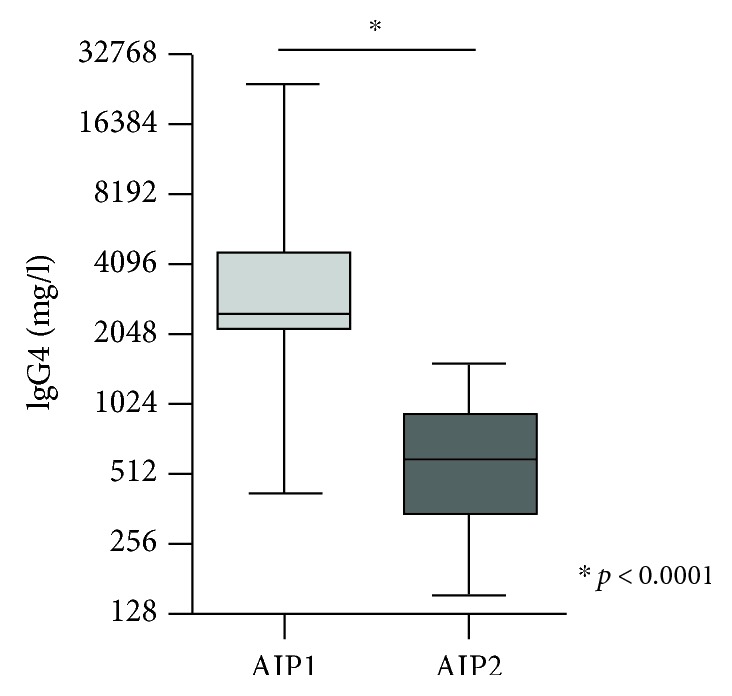
Serum levels of IgG4 are significantly increased in patients with AIP1.

AIP1 patients predominantly revealed a cholestatic course when firstly diagnosed (median bilirubin in AIP1 2.3 mg/dl versus 0.5 mg/dl in AIP2, *p* < 0.001; median alkaline phosphatase (AP) in AIP1 264.0 U/l versus 75.0 U/l in AIP2, *p* < 0.05; and finally, median γ-glutamyltransferase (γ-GT) in AIP1 337.0 U/l versus 37.5 U/l in AIP2, *p* < 0.05; Figures 3(a), 3(b), and 3(c)). There were no significant differences regarding pancreatic enzymes between AIP1 and AIP2 patients (median amylase in AIP1 33.0 U/l and in AIP2 56.5 U/l, *p* = n.s., and median lipase in AIP1 54.0 U/l and in AIP2 75.7 U/l, *p* = n.s.). Patients suffering from AIP1 had significantly higher IgG4 levels (median IgG4 level 2.645 mg/l) while median IgG4 levels in AIP2 patients were within normal ranges with 706.5 mg/l (reference range <1.350 mg/l, *p* < 0.0001; Figure 4).

### 3.3. Genetic Risk Factors

**Figure 5:**
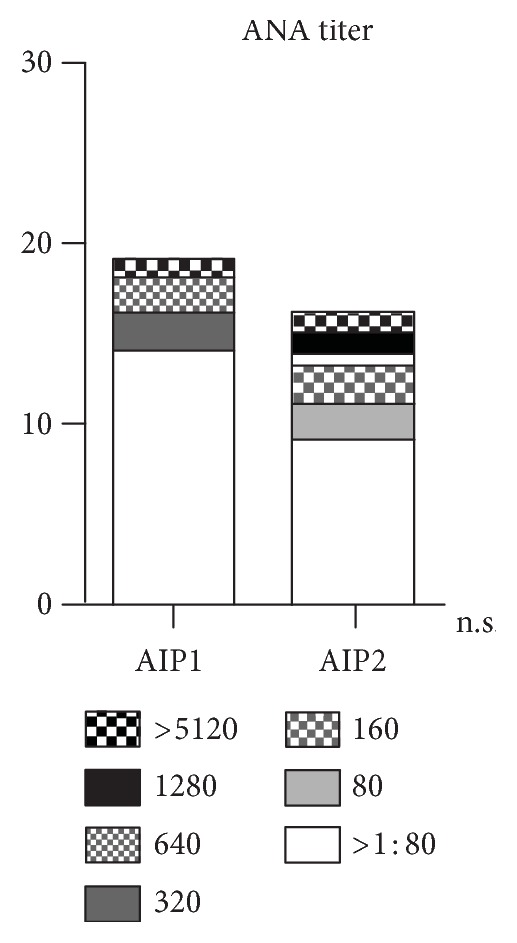
Patients with AIP1 and AIP2 revealed increased ANA titers.

**Figure 6:**
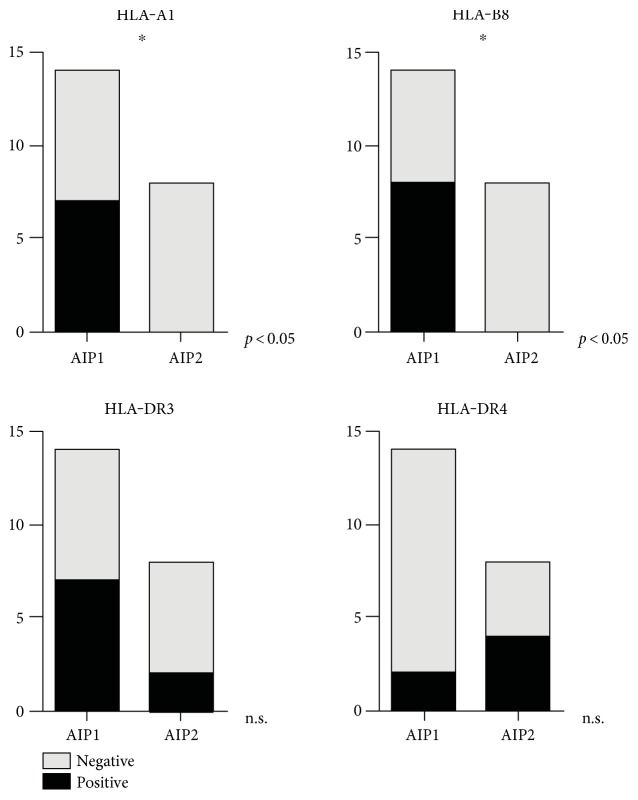
Patients with AIP1 were mainly positive for HLA-B8. However, patients with AIP2 were predominantly positive for HLA-DR4.

We investigated auto-antibody profiles of our cohort testing the following titers: ANA, AMA, ANCA, SMA, LKM, and finally, SLA antibodies. Antibody profiles were available in 19/20 AIP1 patients and in all AIP2 patients. As presented in Figure 5, only 5/19 AIP1 patients and 7/16 AIP2 patients were ANA-positive showing higher titers in the AIP2 group, however missing statistical significance. None of the patients regardless of underlying subtype was positive for the other auto-antibodies as listed previously (data not shown). Human leukocyte antigen (HLA) typing was performed in 22 of 36 patients (14/20 with AIP1 and 8/16 with AIP2) revealing the following results (Figure 6): HLA-A1 was positive in 7/14 patients with AIP1 (50.0%) whereas no patient with AIP2 was positive, *p* < 0.05; HLA-B8 was positive in 8/14 AIP1 patients (57.1%) whereas all AIP2 patients were negative for HLA-B8, *p* < 0.05; HLA-DR3 was positive in 7/14 AIP1 (50.0%) and in 2/8 AIP2 patients (25.0%, *p* = n.s.); and finally, HLA-DR4 was positive in 2/14 AIP1 (14.3%) and in 4/8 AIP2 patients (50.0%, *p* = n.s.). The frequency of HLA-A1, HLA-B8, HLA-DR3, and HLA-DR4 in patients with AIP was compared with the frequency in 1,242,890 ethically matched, healthy controls [17]. Based on the published allele frequencies, HLA phenotype frequencies in healthy controls were determined, resulting in 30.3% for HLA-A1, 21.6% for HLA-B8, 22.9% for HLA-DR3, and finally, 30.7% for HLA-DR4. Thus, patients with AIP1 HLA-A1, HLA-B8, and HLA-DR3 were over-represented, reaching statistical significance (*p* < 0.05) for HLA-B8.

### 3.4. Steroid Response and Immunosuppressive Therapy

All patients initially received steroid-pulse therapy (0.5–1 mg prednisolone/kg/day) to induce remission and were tapered down within 3 months to a maintenance dose of 2.5–7.5 mg prednisolone/kg/day. Response to steroid treatment was excellent in both groups as shown in Table 2 (AIP1 19/20 = 95.0% versus AIP2 15/16 = 93.8%). However, we noticed a high overall rate of relapse during downtapering or maintenance phase (13/36 patients = 36.1%), particularly in AIP1 patients (AIP1 9/20 = 45.0% versus AIP2 4/16 = 25.0%) with the necessity for escalation of the immunosuppressive therapy. Immunosuppression was performed with azathioprine in 7/20 AIP1 (35.0%) and in 2/16 AIP2 (12.5%) patients. Tacrolimus was used in one patient with AIP1 leading to complete remission. However, one patient with AIP1 was treated with 6-mercaptopurine. Finally, in the AIP2 group, one patient received mycophenolate mofetil (MMF) and one patient received rituximab.

### 3.5. Malignancies

We found 3 patients in the AIP1 group who developed CCC. However, in the AIP2 group, only one patient was diagnosed with hepatocellular carcinoma (HCC). None of the patients developed pancreas carcinoma (total survey time 1020 months) (Table 3).

## 4. Discussion

We herein reported on a cohort of 36 Caucasian patients who were diagnosed on the basis of the International Diagnostic Criteria for AIP. In contrast to most studies of the eastern hemisphere, in which up to 90% of the patients suffered from AIP1, we found an almost equal distribution of AIP1 and AIP2 patients (55.6% versus 44.4%) in our cohort. This underlines a fact which recent studies have provided: the incidence of AIP2 is by far more prevalent in Europe and in the United States than in Asia (Table 4) [3, 5, 8, 15, 18–23]. In how far this distribution is based on, the difference of genetic background in AIP patients or due to the limited cohort size has to be further evaluated. Furthermore, a higher frequency of AIP2 patients is reported in surgical series (in our cohort, 10 patients with AIP were diagnosed following surgery) and our hospital is a referral center for inflammatory bowel disease (IBD). These findings might also have an influence on high rates of AIP2 patients in our collective. Moreover, AIP1 patients were significantly older than AIP2 patients (54.4 years versus 40.8 years) and predominantly of male gender (75.0% versus 25.0%) while AIP2 patients were mostly female (68.8% versus 31.2%). These findings reflect the published data of actual literature [5, 24, 25]. Involvement of the biliary tract in sense of AIC was frequent in AIP1 patients with 85.0% (predominantly in terms of intra- and extrahepatic involvement), and these patients consecutively demonstrated a cholestatic course when firstly diagnosed (Figures 3(a), 3(b), and 3(c)). These facts and the significantly higher IgG4 levels in AIP1 patients underline the existing understanding of AIP1 as a pancreatic manifestation of a systemic, IgG4-associated disease with extrapancreatic organ involvement whereas AIP2 only affects the pancreas and rarely shows elevated IgG4 levels [8, 9, 14]. Our study indicates that pancreatic enzymes are not helpful for the diagnosis of AIP. No significant alterations were observed in both types of AIP. Hence, the determination of pancreatic enzymes is not included in the diagnostic algorithm for AIP published by the “International Association of Pancreatology” in 2011 [7].

Compared to other systemic autoimmune diseases like lupus erythematosus, ANA screening with a cut-off greater than 1 : 80 revealed that 5 of our AIP1 patients and 7 of the AIP2 patients were ANA-positive showing higher titers by trend in the AIP2 group. An association of HLA class II alleles with autoimmune conditions is increasingly used for diagnostic purposes. HLA-DR3 and HLA-DR4 are associated with autoimmune hepatitis, and HLA-DR3 is associated with primary sclerosing cholangitis (PSC). We genotyped our cohort and found a trend towards HLA-A1, HLA-B8, and HLA-DR3 positivity for the AIP1 patients and a trend for HLA-DR4 positivity for the AIP2 group. When compared to published allele frequencies in healthy controls, patients with AIP1 HLA-A1, HLA-B8, and HLA-DR3 were over-represented, reaching statistical significance (*p* < 0.05) for HLA-B8. It has to be determined in ongoing studies if HLA typing—as a further brick in the puzzle—might be helpful to subtype patients with IgG4-associated autoimmune pancreatitis.

According to current literature, AIP1 is less likely to be associated with UC than AIP2 [8, 9]. Our study confirms this fact—18.8% of the AIP2 patients suffered from UC while it was only detected in one of the AIP1 patients.

The efficacy of steroid therapy in the treatment of AIP1 and AIP2 has been established by the demonstration of short-term serological (initial response rates > 90%) and radiological responses as well as clinical improvement in several studies. Therefore, it is included as a diagnostic criterion for AIP. Initiation of prednisolone with 0.5–1 mg/kg/day followed by a steroid dose taper has been demonstrated to be highly effective [7, 26–34]. For the initial oral prednisolone dose for induction of remission, 0.5 to 1.0 mg/kg/day is usually used and gradually tapered [26–29, 35]. There is no clear consensus about the duration of steroid therapy, and it is still debated whether steroid maintenance therapy provides beneficial outcomes after remission. In this regard, the Japanese Clinical Guidelines for AIP recommends a steroid maintenance therapy of 5.0 to 7.5 mg prednisolone per day for 3 years after remission [29]. In our cohort, response to steroid treatment was excellent likewise (AIP1 95.0% and AIP2 93.8%). However, relapse rates during steroid taper, maintenance, or offset phases are substantial and vary between 10% and 56%, predominantly affecting AIP1 patients [5, 10, 28, 31–33, 36–40]. Although there is a general agreement that steroids are the ideal initial treatment, no clear consensus regarding treatment for disease relapse exists. According to actual literature, readministration of steroid pulse therapy or addition of immunomodulators such as thiopurine, mycophenolate, rituximab, or tacrolimus is recommended [5, 6, 12, 36, 41–43]. An international multicenter analysis revealed that risk for relapse is higher in AIP1 than in AIP2 patients (31% versus 9%) [5]. Obstructive jaundice, bile duct strictures, diffuse pancreatic swelling, and a sustained high-serum IgG4 level have been reported to be predictors of a relapse [44–47]. A recently published study by Shimizu et al. indicates that the rate of decrease in serum IgG4 level may be a useful predictor of relapse in AIP after steroid therapy [48]. Generally, in 36.1% of our cases, we noticed a relapse during steroid downtapering or maintaining phase, requiring a reapplication of steroids or the addition of further immunomodulatory drugs. In our cohort, sustained clinical, serological, and biochemical responses could be achieved by first-line use of azathioprine (9/13 cases). However, application of tacrolimus, mycophenolate, 6-MP, and rituximab was required in one patient, respectively (Table 2). B-cell deletion therapy with rituximab, a monoclonal antibody directed against the CD20 antigen on B cells, has been effectively used in patients with AIP type 1 as demonstrated by Hart et al. [36]. Interestingly, one patient suffering from AIP2 was successfully treated with rituximab, which was primarily administered for the treatment of concurrent rheumatoid arthritis. As described in current literature, relapsers were more common in AIP1 (45.0%) than in AIP2 (25.0%) patients.

The risk of malignancy in patients with AIP is still debated controversially. It is suggested that patients may be at risk for developing malignancies since AIP shares a number of clinical, biochemical, and imaging features with pancreas carcinoma and cholangiocarcinoma [49–51]. Interestingly, in our cohort with a cumulative survey time of 1020 months in total, 3 of 20 AIP1 patients (15%) developed cholangiocellular carcinoma (CCC) and one of these patients was diagnosed synchronously with AIP. A potential hypothesis of these findings might be that chronic bile duct inflammation and immunosuppressive therapy may increase the risk of developing CCC. One of the 16 AIP2 patients was diagnosed with hepatocellular carcinoma (HCC) synchronously. Remarkably, none of the patients developed pancreas carcinoma during the total survey time. However, drawing conclusions regarding the risk of developing malignancies on the basis of our findings is certainly limited, since patient number was relatively low and overall follow-up was too short.

## 5. Conclusions

In contrast to most studies in Asia in which up to 90% of the patients suffered from AIP1, we noticed a high rate of AIP2 patients in our cohort in Germany with 44.4%. It is still unknown if the higher infection rate with HP may be a possible triggering agent of AIP1 in the eastern hemisphere. AIP1 patients seem to be more frequently older men whereas AIP2 patients are younger and predominantly of female gender. Furthermore, AIP1 patients displayed other organ involvements, especially of the biliary tract (85.0%), whereas AIP2 often suffered from concurrent UC (18.8%). Response to steroid treatment was excellent in patients with AIP1 and AIP2, but disease relapses were also common in both groups.

More multicenter studies with higher number of patients are urgently needed in western countries to define differences and similarities to Asian patients with autoimmune pancreatitis. We therefore hope that our findings may provide a further brick to the puzzle and initiate further studies.

## Figures and Tables

**Table 1:** Demographic and clinical characteristics of our study population with AIP1 and AIP2 (n.s. = not significant) [BMI, body mass index; AIC, autoimmune cholangitis; UC, ulcerative colitis].

Parameter	AIP1	AIP2	*p* value
Number of patients	20 (55.6%)	16 (44.4%)	—
Origin	All Caucasian	All Caucasian	—
Gender (M/F)	15/5	5/11	—
Age (years)	54.4 ± 4.3	40.8 ± 2.7	<0.05
BMI (kg/m^2^)	24.9 ± 1.4	27.0 ± 2.4	n.s.
AIC	17/20 (85.0%)	0/16 (0%)	<0.01
UC	1/20 (5.0%)	3/16 (18.8%)	n.s.

**Table 2:** Immunosuppression and relapse data of patients with AIP1 and AIP2.

Parameter	AIP1	AIP2
Steroid-induced remission	19/20 (95.0%)	15/16 (93.8%)
Relapse	9/20 (45.0%)	4/16 (24%)
Immunosuppression	7/20 (35.0%) Azathioprine	2/16 (12.5%) Azathioprine
	1/20 (5.0%) Tacrolimus	1/16 (6.25%) Mycophenolate
	1/20 (5.0%) 6-Mercaptopurine	1/16 (6.25%) Rituximab

**Table 3:** Incidence of malignancy in our cohort [CCC, cholangiocellular carcinoma; HCC, hepatocellular carcinoma].

Malignancy	Age (years)	Type	Gender	Time
CCC	57	1	F	Metachronously
CCC	76	1	M	Synchronously
CCC	42	1	F	Metachronously
HCC	72	2	M	Synchronously

**Table 4:** Distribution of AIP1 and AIP2 patients in the western hemisphere according to current literature.

	Year	Region	Number of patients	AIP1	AIP2
Sah et al.	2010	US	97	78 (80%)	19 (20%)
Maire et al.	2011	France	44	28 (64%)	16 (36%)
Detlefsen et al.	2012	Denmark	114	63 (55%)	51 (45%)
Hart et al.	2013	US + Japan	1064	978 (92%)	86 (8%)
Ikeura et al.	2013	Italy	92	59 (78%)	17 (22%)
Fritz et al.	2014	Germany	72	40 (56%)	32 (44%)
Bujs et al.	2015	Netherlands	107	96 (90%)	11 (10%)
Rasch et al.	2016	Germany	53	33 (62%)	20 (38%)
López et al.	2016	Spain	52	45 (87%)	7 (13%)

## Abbreviations

AIC: Autoimmune cholangitis
AIP: Autoimmune pancreatitis
AIP1: Autoimmune pancreatitis type 1
AIP2: Autoimmune pancreatitis type 2
AP: Alkaline phosphatase
CCC: Cholangiocellular carcinoma
EUS: Endoscopic ultrasound-guided sonography
GELs: Granulocytic epithelial lesions
γ-GT: γ-Glutamyltransferase
HCC: Hepatocellular carcinoma
HLA: Human leukocyte antigen
IBD: Inflammatory bowel disease
IDCP: Idiopathic duct centric pancreatitis
IgG4: Immunoglobulin G4
LPSP: Lymphoplasmacytic sclerosing pancreatitis
MMF: Mycophenolate mofetil
UC: Ulcerative colitis.
